# Preliminary comparison of three-dimensional reconstructed palatal morphology in subjects with different sagittal and vertical patterns

**DOI:** 10.1186/s12903-020-1041-9

**Published:** 2020-02-17

**Authors:** Xiaoyi Huang, Xinnong Hu, Yijiao Zhao, Yong Wang, Yan Gu

**Affiliations:** 1 Department of orthodontics, Peking University School and Hospital of Stomatology, No. 22 Zhongguancun Avenue South, Haidian District, Beijing, 100081 China; 2 National Engineering Laboratory for Digital and Material Technology of Stomatology, Beijing Key Laboratory of Digital Stomatology, Peking University School and Hospital of Stomatology, No. 22 Zhongguancun Avenue South, Haidian District, Beijing, 100081 China

**Keywords:** Skeletal class II, Retrusive mandible, Vertical pattern, Three-dimension, Palatal morphology

## Abstract

**Background:**

The aim of this study was to assess the difference of palatal morphology in different vertical patterns between skeletal Class I subjects and skeletal Class II subjects with retrusive mandible.

**Methods:**

Seventy-six skeletal Class II subjects with retrusive mandible (38 females, 38 males) and 85 skeletal Class I subjects (45 females, 40 males) were collected retrospectively and divided into hyperdivergent, normodivergent and hypodivergent groups. CBCT images of these subjects were reoriented by Dolphin 3D Imaging software. Three-dimensional (3D) maxilla was segmented by ProPlan software before using Geomagic Studio software to reconstruct 3D palatal morphology. Deviation patterns on 3D colored map analysis was performed to compare the difference of 3D palatal morphology between different groups.

**Results:**

3D colored map analysis showed that male’s palate was higher and wider than that of female in the posterior part, regardless of different sagittal and vertical patterns. In skeletal Class II subjects with retrusive mandible, males with hyperdivergent and normodivergent showed higher and narrower in the posterior part of palate, while females with hyperdivergent and normodivergent had a higher but no obviously narrow palate compared with the hypodivergent subjects. Skeletal Class II subjects with retrusive mandible showed flatter and narrower in the posterior part of palate than that of skeletal Class I subjects.

**Conclusions:**

Sagittal and vertical patterns have great influence on the palatal morphology and as the vertical dimension increased, the palate tended to be higher and narrower.

## Background

Previous studies with traditional methods to analyze cephalograms and observe dental casts focus on the influence of sagittal pattern on palatal morphology and suggested that skeletal Class II subjects was associated with narrow and high palatal morphology [1–3], especially those with retrusive mandible. However, literatures about the influence of vertical pattern on palatal morphology are insufficient and even controversial [4–6]. Three-dimensional (3D) methods to compare palatal morphology included either directly measuring palatal width, height, area and volume on cone-beam computed tomography (CBCT) images or dental casts or obtaining 3D coordinates of palate, and then established two-dimensional (2D) mathematical models or superimposed on palate for comparison [7–12]. Direct measurement to reflect the differences between groups is convenient but insufficient due to different display of specific structure of palate and thus incorrect interpretation of the results influenced by tooth position and inclination. Utilizing 3D coordinates to establish 2D mathematical models or superimpose on 3D palatal morphology can reflect more morphologic information of palate and show the differences more intuitively, but it is very complicated and still subject to the specific landmarks of palate. Use of geometric morphometric method (GMM) to visually compare palatal morphology has been the focus of recent studies [4, 5, 10, 11]. For example, Laganà et al. used GMM to reveal the palatal shape variation between open bite subjects and control subjects, which obtained a total of 239 3D coordinate points on digital maxillary casts, and applied procrustes superimposition for palatal shape description, thus performed principal component analysis to find the major factors to influence the morphology of palate [10].

However, with the development of digital technology, digital software (e.g. ProPlan (Materialise, Leuven, Belgium) and Geomagic (Durham, NC, US) software can be used to reconstruct 3D morphology [12, 13]. The aim of this study was to use digital software to reconstruct 3D palatal morphology in skeletal Class II subjects with retrusive mandible and skeletal Class I subjects, then describe palatal morphology in different sagittal and vertical patterns.

## Methods

### Subjects

Seventy-six skeletal Class II subjects with retrusive mandible (38 females, 38 males; mean age 25.63 ± 4.76 years) and 85 skeletal Class I subjects (45 females, 40 males; mean age 23.95 ± 4.45 years) were collected retrospectively from Department of Oral Maxillofacial Surgery and Department of Orthodontics, Peking University School and Hospital of Stomatology. Biomedical Ethics Committee of Peking University School and Hospital of Stomatology has approved this study (Number: PKUSSIRB-201946086). Written informed consent was obtained from all participants included in the study.

### Inclusion and exclusion criteria

The inclusion criteria for skeletal Class II subjects with retrusive mandible were the following: (1) Mongolian, (2) aged 18–35 years, (3) 78.8° < SNA < 86.8°, SNB < 76.2°, ANB > 4.7°, NP-FH < 81.7° (according to Chinese cephalometric norms), (4) no previous orthodontic or orthognathic treatment. Exclusion criteria for skeletal Class II with retrusive mandible included: (1) missing permanent teeth, (2) retained deciduous teeth, (3) impacted teeth, (4) severe periodontitis, (5) history of palatal surgery, (6) cleft lip and/or palate, (7) craniofacial syndromes. According to Chinese cephalometric norms, skeletal Class I subjects with 78.8° < SNA < 86.8°, 76.2° < SNB < 84°, 0.7° < ANB < 4.7°, and 81.7° < NP-FH < 89.1° were enrolled in this study. The other inclusion criteria and exclusion criteria for skeletal Class I subjects were the same as those for skeletal Class II subjects with retrusive mandible.

### Groups

According to the values of SN-MP, FH-MP and S-Go/N-Me, skeletal Class II subjects with retrusive mandible and skeletal Class I subjects were divided into the following sub-groups:

Hyperdivergent: SN-MP > 37.7°, FH-MP > 32°, S-Go/N-Me< 62%;

Normodivergent: 27.3° < SN-MP < 37.7°, 22° < FH-MP < 32°, 62% < S-Go/N-Me< 68%;

Hypodivergent: SN-MP < 27.3°, FH-MP < 22°, S-Go/N-Me> 68%.

Descriptions of six groups (Class II-hype, Class II-norm, Class II-hypo, Class I-hype, Class I-norm, and Class I-hypo) were shown in Table 1.

**Table 1:** Demographic Data of the Subjects

	Class II-hype		Class II-norm		Class II-hypo		Class I-hype		Class I-norm		Class I-hypo	
	Male	Female	Male	Female	Male	Female	Male	Female	Male	Female	Male	Female
n	15	15	15	15	8	8	11	15	14	15	15	15
Age(y)	25.60 ± 5.89	25.47 ± 4.37	26.67 ± 5.04	23.60 ± 3.94	26.38 ± 4.66	28.50 ± 2.38	22.58 ± 3.15	25.87 ± 4.55	22.29 ± 4.23	22.60 ± 3.18	24.80 ± 5.13	25.20 ± 4.97
SNA(°)	82.21 ± 2.12	81.09 ± 2.18	81.44 ± 2.30	81.55 ± 1.30	80.63 ± 0.91	83.05 ± 2.29	80.35 ± 1.11	82.05 ± 1.87	81.51 ± 2.20	81.70 ± 1.88	82.02 ± 2.20	81.82 ± 2.02
SNB(°)	73.08 ± 2.14	71.95 ± 2.51	74.00 ± 1.65	74.45 ± 1.56	74.76 ± 0.89	74.90 ± 1.13	77.46 ± 0.49	78.55 ± 1.57	78.95 ± 1.92	79.13 ± 1.50	79.21 ± 2.17	79.11 ± 2.09
ANB(°)	9.11 ± 1.94	9.17 ± 1.82	7.38 ± 1.39	7.10 ± 1.45	5.86 ± 0.83	8.13 ± 1.36	2.89 ± 1.31	3.49 ± 0.85	2.56 ± 1.18	2.53 ± 1.16	2.81 ± 1.01	2.71 ± 1.12
NP-FH(°)	79.47 ± 2.76	80.00 ± 1.99	81.10 ± 0.85	81.49 ± 0.14	81.41 ± 0.29	81.60 ± 0.14	86.23 ± 2.17	85.99 ± 1.80	86.09 ± 1.28	87.62 ± 1.45	87.59 ± 1.34	87.49 ± 1.34
SN-MP(°)	42.83 ± 3.89	45.00 ± 4.68	33.29 ± 1.82	34.07 ± 2.07	22.53 ± 3.32	24.63 ± 1.78	39.92 ± 2.38	39.82 ± 2.35	31.61 ± 2.27	32.33 ± 2.49	23.03 ± 3.42	23.16 ± 4.06
FH-MP(°)	40.07 ± 3.33	40.54 ± 4.21	29.87 ± 1.66	30.33 ± 1.49	19.93 ± 3.07	20.98 ± 1.16	34.65 ± 2.01	35.39 ± 2.74	28.71 ± 2.06	28.03 ± 2.63	19.04 ± 2.66	18.79 ± 3.72
S-Go/N-Me(%)	60.36 ± 2.12	57.63 ± 3.02	66.98 ± 1.35	65.72 ± 1.59	75.63 ± 3.10	72.88 ± 1.19	60.71 ± 1.84	60.11 ± 2.13	67.01 ± 1.65	66.11 ± 1.42	73.90 ± 3.31	73.55 ± 4.02

*SNA* Angle formed by sella–nasion–A-point, *SNB* angle formed by sella–nasion–B-point, *ANB* angle formed by A-point–nasion–B-point, *NP-FH* angle between the nasion-pogonion line and frankfort horizontal plane, *SN-MP* angle between the sella-nasion line and mandibular plane, *FH-MP* angle between frankfort horizontal plane and mandibular plane, *S-Go/N-Me* ratio of the distance of sella-gonion to the distance of the nasion-menton

Values are presented as mean ± standard deviation

### CBCT

NewTom Scanner (NewTom AG, Marburg, Germany) was used to take CBCT images for the subjects. All images were taken with 0.3-mm axial slice thickness, 15 × 15-cm field of view, 3.6-s scan time, 110-kV tube voltage, and 2.81-mA tube current. CBCT were exported in digital imaging and communications in medicine (DICOM) format [14]. Lateral cephalograms were generated from CBCT images by Dolphin 3D Imaging software (version 11.8, Dolphin Imaging and Management Solutions, Chatsworth, Calif).

**Fig. 1:**
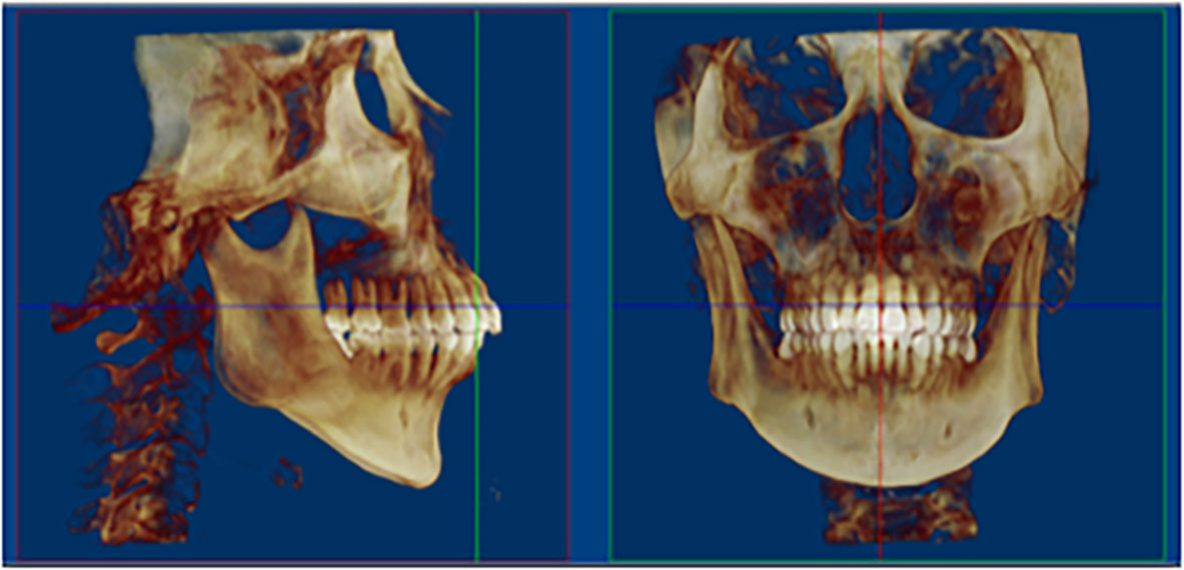
Reorientation for reconstruction of three-dimensional (3D) palatal morphology. Horizontal plane, the plane tangent to the most inferior slice of the maxillary alveolar bone; sagittal plane, the plane perpendicular to the horizontal plane, passing through the ANS-PNS line; coronal plane, the plane perpendicular to the above two planes

To set identical 3D reference planes in different groups, CBCT images were reoriented and exported by Dolphin software to obtain palatal morphology. Horizontal plane was the plane tangent to the most inferior slice of maxillary alveolar bone. Sagittal plane was the plane that passed through ANS-PNS line and was perpendicular to horizontal plane. Coronal plane was the plane perpendicular to the above two planes [8, 15] (Fig. 1).

### 3D palatal morphology reconstruction and measurements

**Fig. 2:**
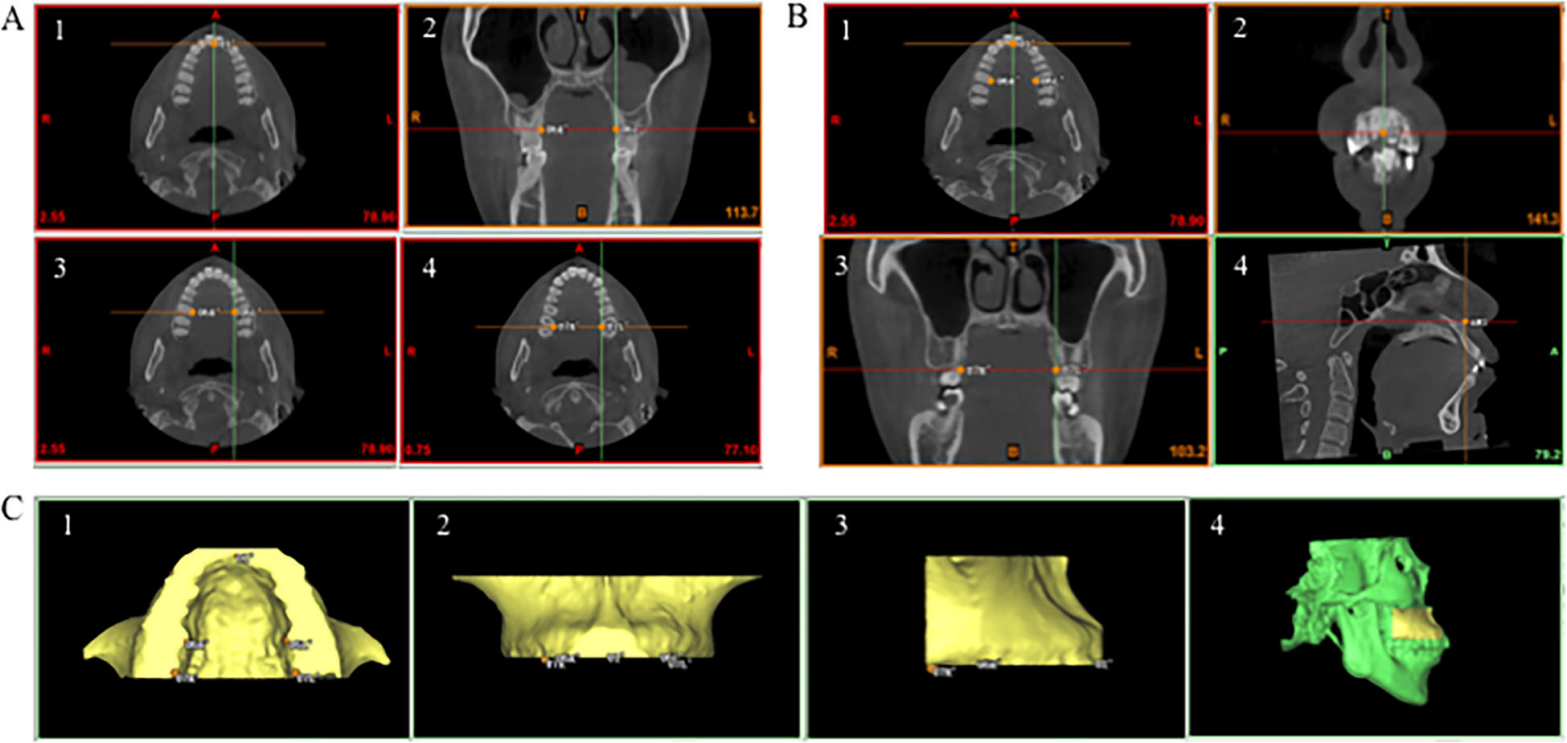
3D maxilla was separated from the 3D skull. **a**, The landmarks used to reconstruct the palatal morphology. 1, U1’ in the horizontal plane; 2 and 3, U6L’ and U6R’ in the coronal and horizontal plane; 4, U7L’ and U7R’ in the horizontal plane. **b**, The boundary of the maxilla. 1, The lowermost horizontal plane was through the U1’, U6L’ and U6R’; 2, the foremost coronal plane was through the U1’; 3, the backmost coronal plane was through the U7L’or U7R’; 4, the uppermost horizontal plane was through the ANS. **c**, The 3D objects of maxilla. 1, Upwards view; 2, front view; 3, lateral view; 4, the maxilla in the skull

**Fig. 3:**
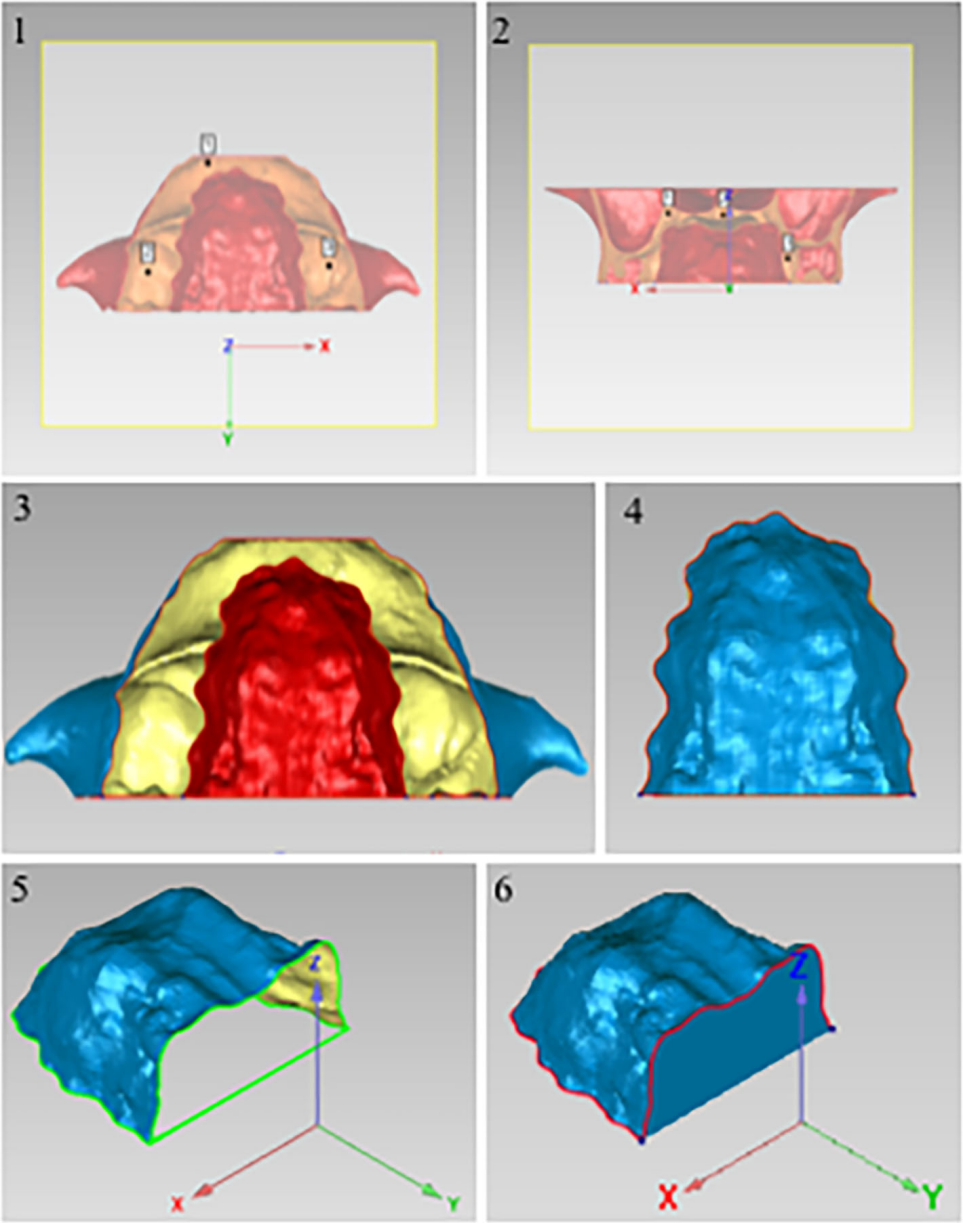
Reconstruction of 3D palatal morphology. 1, The plane cutting of the lowermost horizontal plane of the maxilla; 2, the plane cutting of the backmost coronal plane of the maxilla; 3, the palate was selected to create a bounded component; 4, the palate was remained and the residual sections were removed; 5 and 6, the 3D closed figure of the palate was obtained by filling the boundary hole

Reoriented CBCT images were imported into ProPlan CMF 1.4 software (Materialise, Leuven, Belgium) for 3D reconstruction [16]. The landmarks are defined in Table 2 and Fig. 2a. Firstly, 3D skull was obtained by threshold segmentation. To separate maxilla from 3D skull, the boundary of maxilla was defined by the following specific planes: the lowermost horizontal plane was through U1’, U6L’ and U6R’; the foremost coronal plane was through U1’, the backmost coronal plane was through U7L’or U7R’; and the uppermost horizontal plane was through ANS (Fig. 2b). Then 3D shape of maxilla was segmented and exported as standard tessellation language (STL) format document (Fig. 2c). To transfer reconstructed 3D object of maxilla into 3D palatal morphology, STL format documents were imported into Geomagic Studio 11.0 software (Raindrop Geomagic, Inc., NC, USA). Through the method of plane cutting, the lowermost horizontal plane and backmost coronal plane of maxilla were used to separate palate from maxilla, and then the palate was selected to create a bounded component, and the residual sections were removed. Finally, 3D closed figure of palate was obtained by filling the boundary hole (Fig. 3).

**Table 2:** Definition of Landmarks for 3D Palatal Morphology

Landmark	Definition
U1’	The lowest point on the temporal side of the maxillary anterior alveolar ridge on the median sagittal plane
U6L’	The lowest point on the middle of the temporal alveolar ridge of the left maxillary first molar
U6R’	The lowest point on the middle of the temporal alveolar ridge of the right maxillary first molar
U7L’	The farthest and lowest point on the temporal alveolar ridge of the left maxillary second molar
U7R’	The farthest and lowest point on the temporal alveolar ridge of the right maxillary second molar

All landmarks in Table 2 are shown in Fig. 2

**Fig. 4:**
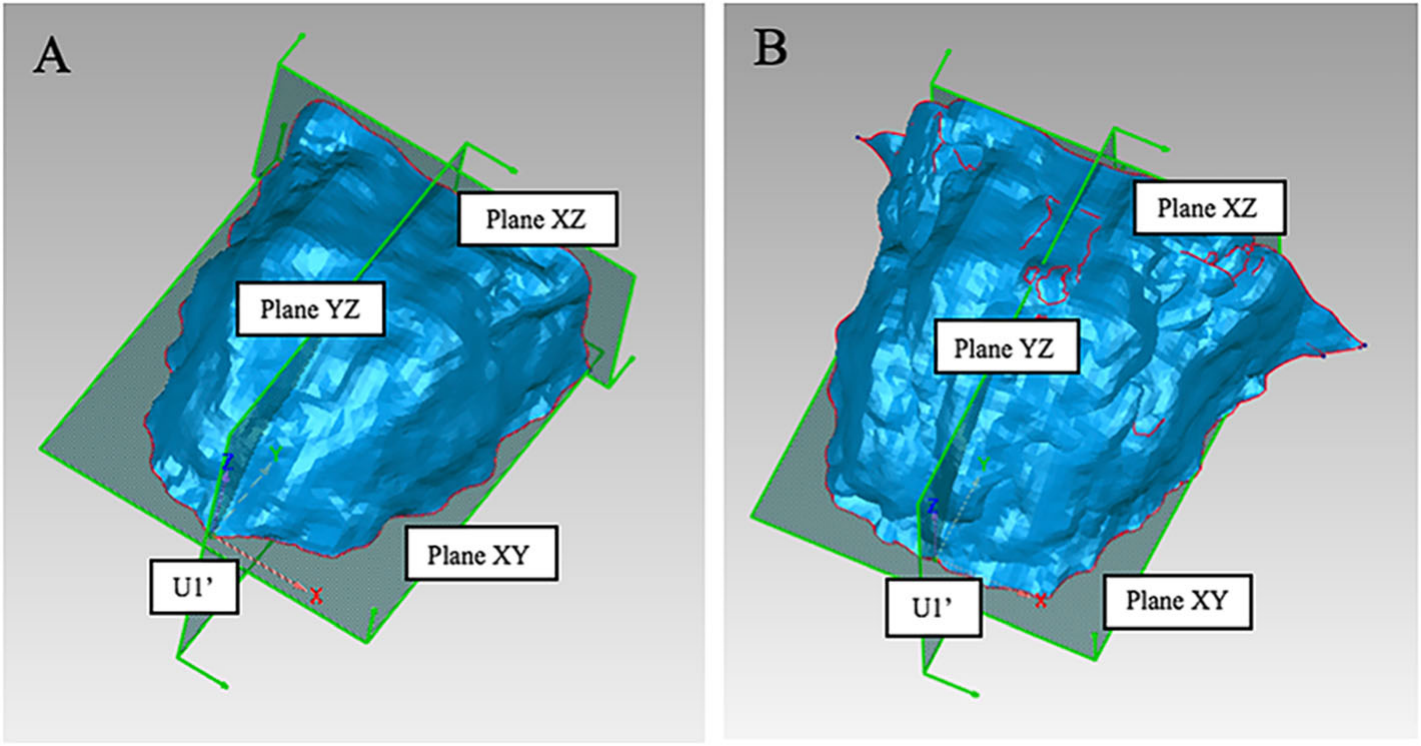
3D coordinate system. **a**, The three-dimensional (3D) coordinate system of the palatal morphology. U1’, The origin; Plane XY, the horizontal plane; Plane YZ, the sagittal plane; and Plane XZ, the coronal plane. **b**, The average 3D palatal morphology for each group established by the method of average calculation

In order to compare palatal morphology between different groups, it is necessary to obtain the average morphology of each group and put them into the same 3D coordinate system. U1’ was set as the origin; horizontal plane passing through U1’ was set as plane XY; sagittal plane passing through U1’ was set as plane YZ; and the backmost coronal plane perpendicular to the above two planes was set as plane XZ (Fig. 4a). All 3D palatal models were put into this coordinate system and then an average 3D palatal morphology for each group were established with the method of average calculation (Fig. 4b).

**Fig. 5:**
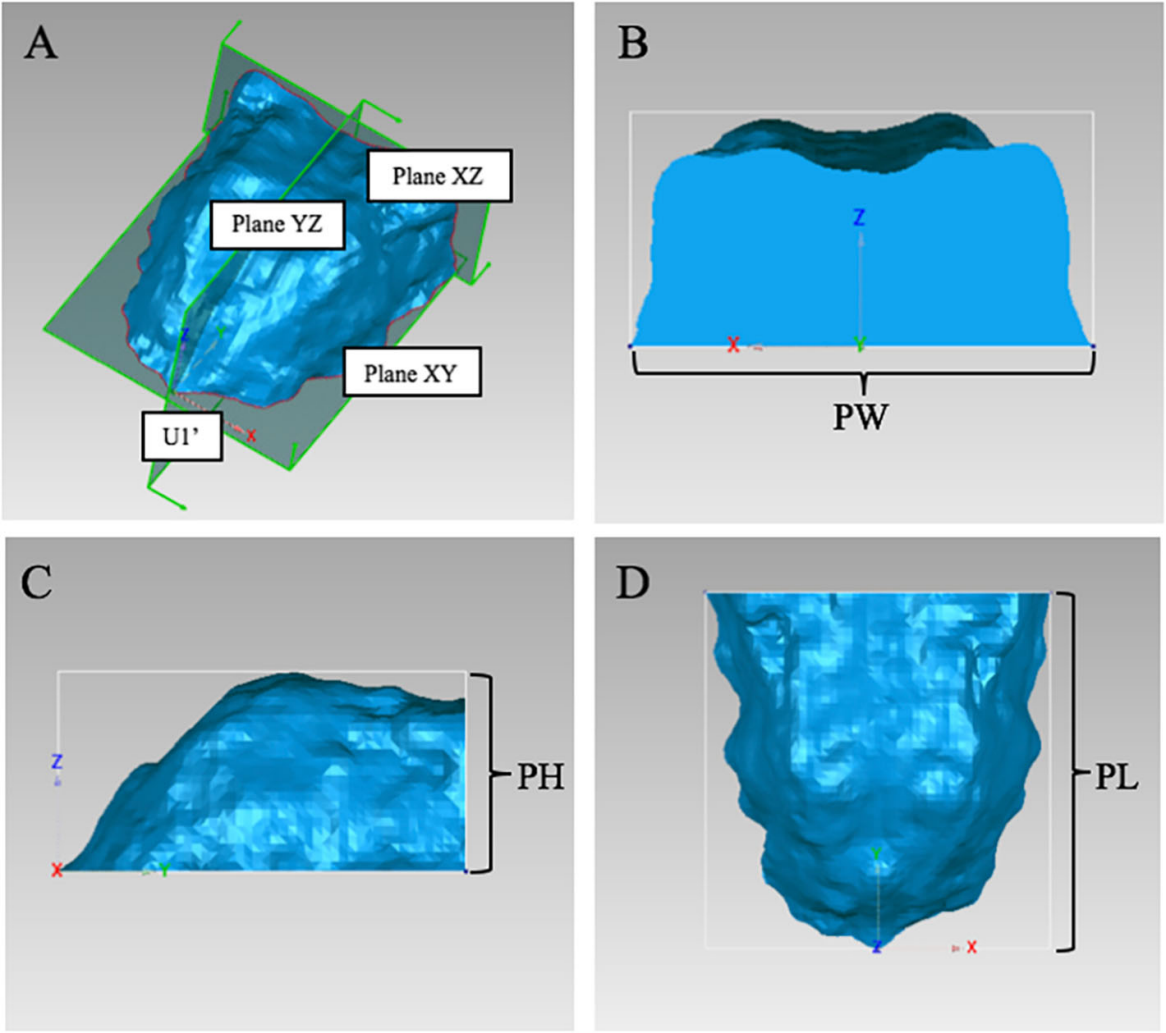
Measurements of 3D palatal morphology. **a**, Palatal volume (PV) and palatal area (PA) were calculated using Geomagic studio. **b**, Palatal width (PW) was measured as the width of the bounding box. **c**, Palatal height (PH) was measured as the height of the bounding box. **d**, Palatal length (PL) was measured as the length of the bounding box

Palatal volume (PV) was calculated as the volume of 3D closed figure of palate, and palatal area (PA) was calculated as the surface area of 3D closed figure of palate using Geomagic studio (Fig. 5a). Besides, palatal width (PW) was measured as the width of the bounding box, palatal height (PH) was measured as the height of the bounding box, and palatal length (PL) was measured as the length of the bounding box using Geomagic studio (Fig. 5b, c, d) [4].

### Statistical analysis

To determine inter- and intra-observer reliability of this method, 20 CBCT images were randomly selected and 3D palatal shape was re-established by two authors (Xiaoyi, Huang and Xinnong, Hu) with 2-week interval. Pearson’s correlation was performed to calculate intraclass correlation coefficient (ICC). The comparison of 3D palatal morphology among different groups was evaluated through deviation patterns on 3D colored map analysis using Geomagic studio. Root mean square estimate values (RMSE) was used to assess the difference values in the comparison of 3D palatal morphology between different vertical pattern groups with Geomagic studio. Independent 2-sample *t* test was used to analyze the differences of PV and PH among different sagittal and vertical pattern groups. All statistics were performed by SPSS software ver. 23.0 (IBM, Armonk, NY, USA).

## Results

ICC values of PV, PA, PW, PH and PL were larger than 0.80 (Table 3), indicating good inter- and intra-observer reliability of this 3D reconstruction method.

**Table 3:** Intraclass Correlation Coefficients (ICCs) for Measurements of 3D Palatal Morphology

	Interobserver	Intraobserver
PV	0.890	0.926
PA	0.836	0.879
PW	0.852	0.918
PH	0.946	0.959
PL	0.867	0.837

*PV* palatal volume, *PA* palatal area, *PW* palatal width, *PH* palatal height, *PL* palatal length

### Gender difference of 3D palatal morphology

#### Skeletal class II subjects with retrusive mandible

**Fig. 6:**
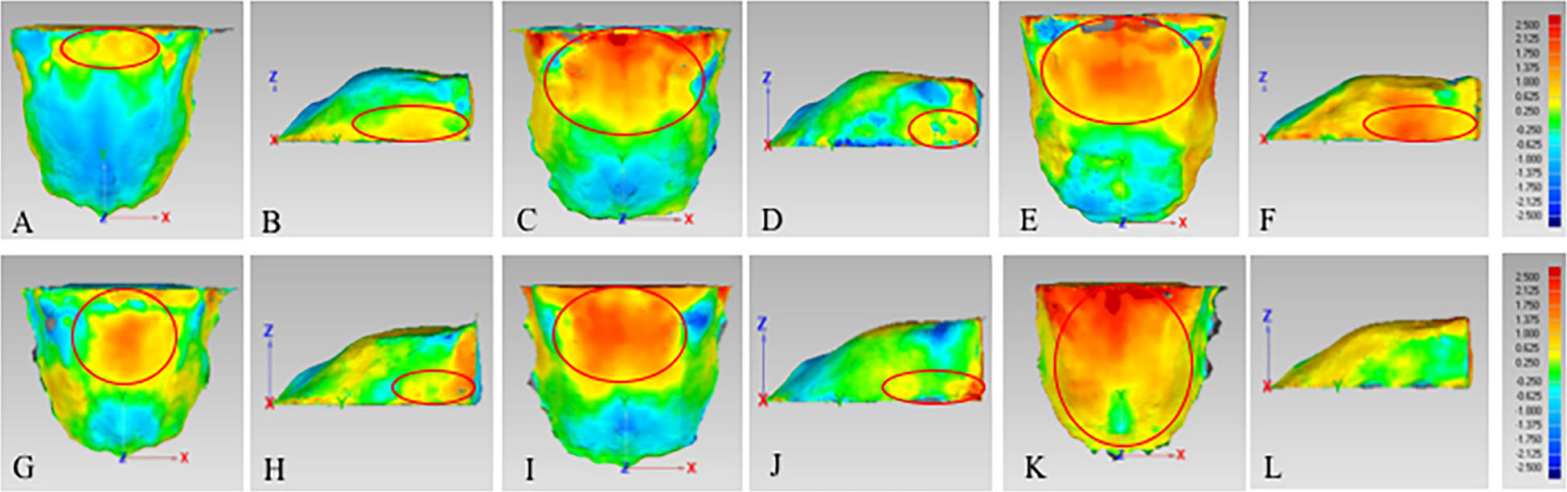
Comparison of 3D palatal morphology in different genders. Deviation within 0.25 mm marked in green, ≥ 2.50 mm marked in red, ≤ -2.50 mm marked in dark blue. Red circle represents markedly positive deviation. Positive deviation means male’s palate was larger than the female’s. **a** and **b**, The deviation pattern in Class II-hype group; **c** and **d**, the deviation pattern in Class II-norm group; **e** and **f**, the deviation pattern in Class II-hypo group; **g** and **h**, the deviation pattern in Class I-hype group; **i** and **j**, the deviation pattern in Class I-norm group; **k** and **l**, the deviation pattern in Class I-hypo group

In Class II-hype group, Class II-norm group and Class II-hypo group, the posterior part of male’s palate was higher than that of female, and the difference were 0.63–1.00 mm, 0.63–2.50 mm and 0.63–2.50 mm, respectively (Fig. 6a, c, e). As for the width of the palate, male’s palate was wider than that of female in the posterior part in three groups, and the difference were 0.63–1.00 mm, 0.63–1.38 mm and 0.63–2.13 mm, respectively (Fig. 6b, d, f).

#### Skeletal class I subjects

In three groups, male’s palate was higher than that of female in the posterior part (Fig. 6g, i, k). As for the width of palate, male’s palate was wider than that of female in the posterior part in Class I-hype and Class I-norm groups, and the difference were 0.63–1.38 mm, while Class I-hypo group showed no remarkable difference in the posterior part between the males and females, with the difference less than 0.25 mm (Fig. 6h, j, l).

### Comparison of 3D palatal morphology with different vertical patterns

#### 3D palatal morphology in class II groups

**Fig. 7:**
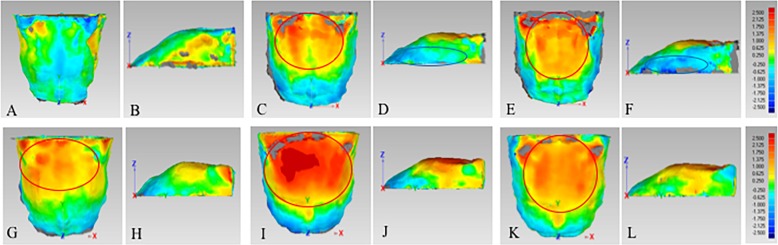
Comparison of 3D palatal morphology in skeletal Class II subjects with different vertical patterns. Deviation within 0.25 mm marked in green, ≥ 2.50 mm marked in red, ≤ -2.50 mm marked in dark blue. Red circle represents markedly positive deviation, while blue circle represents markedly negative deviation. **a**-**f**, The deviation pattern in male. **g**-**l**, The deviation pattern in female. **a**, **b**, **g** and **h**, Positive deviation means palate of Class II-hype subjects was larger than Class II-norm subjects, while negative deviation means palate of Class II-hype subjects was smaller than Class II-norm subjects. **c**, **d**, **i** and **j**, Positive deviation means palate of Class II-hype subjects was larger than Class II-hypo subjects, while negative deviation means palate of Class II-hype subjects was smaller than Class II-hypo subjects. **e**, **f**, **k** and **l**, Positive deviation means palate of Class II-norm subjects was larger than Class II-hypo subjects, while negative deviation means palate of Class II-norm subjects was smaller than Class II-hypo subjects

In males, the height and width of the palate showed no remarkable difference between Class II-hype and Class II-norm groups (Fig. 7a, b). However, the palate of the subjects in Class II-hype and Class II-norm groups were both higher in the posterior part than that of Class II-hypo group, and the difference were similar about 0.63–2.50 mm (Fig. 7c, e). Regarding the width of the palate, it was narrower in Class II-hype and Class II-norm groups than that in Class II-hypo group and the difference was 0.63–1.00 mm and 0.63–1.75 mm, respectively (Fig. 7d, f). The maximum RMSE value for comparison among these three groups was 0.98 mm, which was noted when Class II-norm group was compared with Class II-hypo group (Table 4).

**Table 4:** Deviation of 3D Palatal Morphology Among Different Vertical Pattern Groups

		Class II-hype-Class II-norm	Class II-hype-Class II-hypo	Class II-norm-Class II-hypo	Class I-hype-Class I-norm	Class I-hype-Class I-hypo	Class I-norm-Class I-hypo
Male	Standard deviation (mm)	0.59	0.69	0.98	0.97	0.93	0.48
	RMSE (mm)	0.59	0.69	0.98	1.02	0.99	0.48
Female	Standard deviation (mm)	0.65	1.03	0.71	0.35	0.63	0.55
	RMSE (mm)	0.70	1.16	0.77	0.35	0.70	0.61

*RMSE* root mean square estimate values

In females, the posterior part of the palate in Class II-hype group was higher than that in Class II-norm and Class II-hypo groups (Fig. 7g, i). Additionally, the posterior part of the palate in Class II-norm group was higher than that in Class II-hypo group, with the difference of 0.63–1.75 mm (Fig. 7k). The width of the palate among these three groups showed no significant differences (Fig. 7h, j, l). The maximum RMSE value of 1.16 mm was noted when Class II-hype group was compared with Class II-hypo group (Table 4).

#### 3D palatal morphology in class I groups

**Fig. 8:**
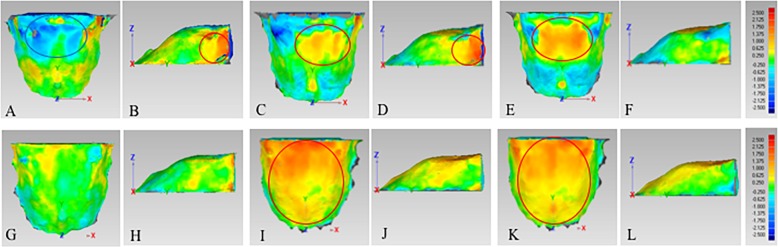
Comparison of 3D palatal morphology in skeletal Class I subjects with different vertical patterns. Deviation within 0.25 mm marked in green, ≥ 2.50 mm marked in red, ≤ -2.50 mm marked in dark blue. Red circle represents markedly positive deviation, while blue circle represents markedly negative deviation. **a**-**f**, The deviation pattern in male. **g**-**l**, The deviation pattern in female. **a**, **b**, **g** and **h**, Positive deviation means palate of Class I-hype subjects was larger than Class I-norm subjects, while negative deviation means palate of Class I-hype subjects was smaller than Class I-norm subjects. **c**, **d**, **i** and **j**, Positive deviation means palate of Class I-hype subjects was larger than Class I-hypo subjects, while negative deviation means palate of Class I-hype subjects was smaller than Class I-hypo subjects. **e**, **f**, **k** and **l**, Positive deviation means palate of Class I-norm subjects was larger than Class I-hypo subjects, while negative deviation means palate of Class I-norm subjects was smaller than Class I-hypo subjects

In males, the posterior part of the palate in Class I-hype group was flatter than that in Class I-norm group with the difference of 0.63–1.75 mm (Fig. 8a). However, the posterior part of the palate in Class I-hype and Class I-norm groups were higher than that in Class I-hypo group, and the difference were 0.63–1.38 mm (Fig. 8c, e). As for the width of the palate, the posterior part in Class I-hype group was wider than that in Class I-norm and Class I-hypo groups with the difference of 0.63–2.13 mm, but no remarkable difference between Class I-norm and Class I-hypo groups was found (Fig. 8b, d, f). The maximum RMSE value of 1.02 mm was observed when Class I-hype group was compared with Class I-norm group (Table 4).

In females, no remarkable difference was noted when compared Class I-hype group with Class I-norm group in palatal height and width (Fig. 8g, h). However, the palate in Class I-hype and Class I-norm groups were both higher than that in Class I-hypo group, and the difference was 0.63–2.13 mm (Fig. 8i, k), while no remarkable difference was noted in palatal width (Fig. 8j, l). The maximum RMSE value of 0.70 mm was noted when Class I-hype group was compared with Class I-hypo group (Table 4).

### Comparison of 3D palatal morphology in different sagittal patterns

**Fig. 9:**
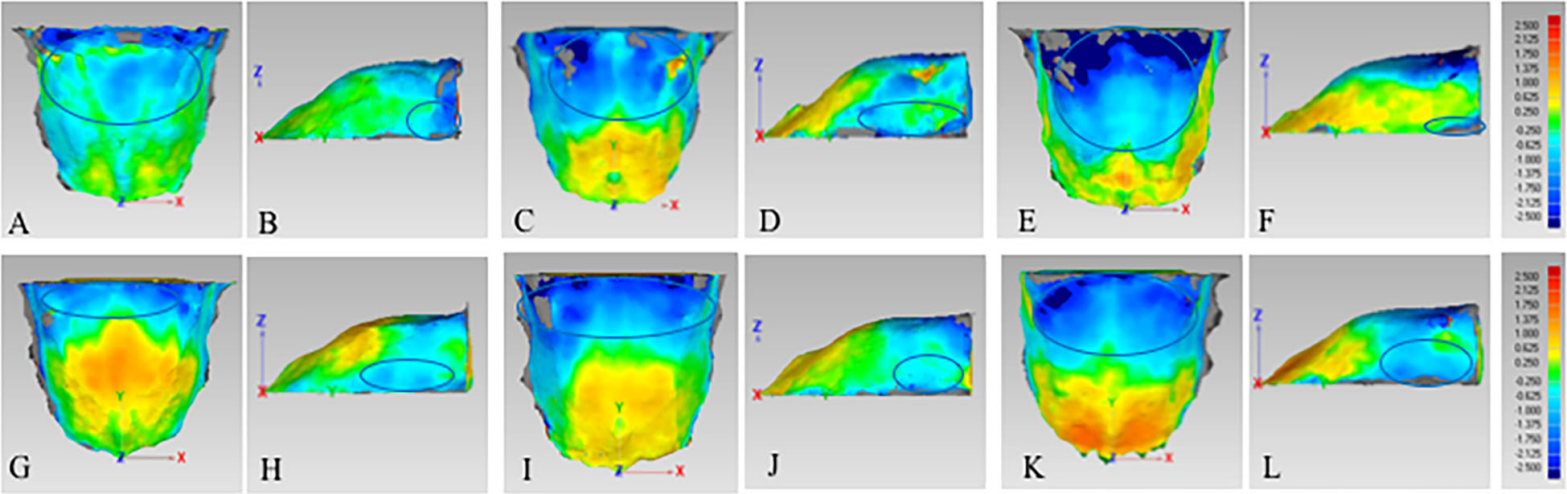
Comparison of 3D palatal morphology in different sagittal patterns. Deviation within 0.25 mm marked in green, ≥ 2.50 mm marked in red, ≤ -2.50 mm marked in dark blue. Blue circle represents markedly negative deviation. **a**-**f**, The deviation pattern in male. **g**-**l**, The deviation pattern in female. **a**, **b**, **g** and **h**, Positive deviation means palate of Class II-hype subjects was larger than Class I-hype subjects, while negative deviation means palate of Class II-hype subjects was smaller than Class I-hype subjects. **c**, **d**, **i** and **j**, Positive deviation means palate of Class II-norm subjects was larger than Class I-norm subjects, while negative deviation means palate of Class II-norm subjects was smaller than Class I-norm subjects. **e**, **f**, **k** and **l**, Positive deviation means palate of Class II-hypo subjects was larger than Class I-hypo subjects, while negative deviation means palate of Class II-hypo subjects was smaller than Class I-hypo subjects

In Class II-hype group, the posterior part of palate was flatter than that in Class I-hype group for both gender, with the difference of 0.63–2.13 mm in males and 0.63–1.75 mm in females (Fig. 9a, g). Furthermore, PH of males in Class II-hype group showed significantly smaller than that in Class I-hype group (*p* < 0.05, Table 5).

**Table 5:** Comparison of Palatal Volume and Palatal Height between Class II and Class I Subjects

		Mean	SD	*p*-value
Male	PV (mm^3^)			
	ClassII-hype	10,903.41	1902.01	0.81
	Class I-hype	11,093.46	2046.11	
	Class II-norm	10,266.46	2416.64	0.13
	Class I-norm	11,517.11	1809.19	
	Class II-hypo	10,574.33	1888.23	0.60
	Class I-hypo	10,950.78	1426.81	
	PH (mm)			
	Class II-hype	14.60	1.59	0.04*
	Class I-hype	16.78	2.78	
	Class II-norm	14.46	2.40	0.02*
	Class I-norm	16.38	1.76	
	Class II-hypo	14.48	2.26	0.16
	Class I-hypo	15.73	1.80	
Female	PV (mm^3^)			
	Class II-hype	10,202.18	1605.50	0.72
	Class I-hype	9947.32	2158.18	
	Class II-norm	9645.65	1240.42	0.95
	Class I-norm	9672.40	1275.10	
	Class II-hypo	8633.69	1207.03	0.65
	Class I-hypo	9046.24	1638.68	
	PH (mm)			
	Class II-hype	14.60	2.42	0.61
	Class I-hype	15.04	2.21	
	Class II-norm	13.41	1.34	0.05*
	Class I-norm	14.52	1.55	
	Class II-hypo	12.05	1.48	0.11
	Class I-hypo	13.74	1.83	

*SD* standard deviation, *PV* palatal volume, *PH* palatal height

*p < 0.05

In Class II-norm group, the posterior part of palate was also flatter than that in Class I-norm group, and the difference was 0.63–2.50 mm (Fig. 9c, i). PH in Class II-norm group showed significantly smaller than that in Class I-norm group (male: *p* < 0.05; female: *p* = 0.05, Table 5).

In Class II-hypo group, the posterior part of palate was flatter than that in Class I-hypo group, with the difference of more than 2.50 mm in males and 0.63–2.50 mm in females (Fig. 9e, k).

As for the width, the posterior part of palate in Class II groups was narrower than that in Class I groups with the similar vertical pattern for males and females (Fig. 9b, d, f, h, j, l). However, comparison of PV between skeletal Class II with retrusive mandible and skeletal Class I subjects showed no significant difference (*p* > 0.05, Table 5).

## Discussion

All CBCT images in our study were collected from the patients records who previously visited Department of Oral Maxillofacial Surgery or Department of Orthodontics. According to the guidelines published by the American Academy of Oral and Maxillofacial Radiology [17], all CBCT images were taken as necessary examination for different purposes of diagnosis and treatment planning, including to confirm whether with potential dental structural anomalies, craniofacial asymmetries, temporomandibular joint problems and so on. Previous studies on comparison of palatal morphology in subjects among different groups were mainly based on 2D measurements on dental casts and cephalometric radiographs [1, 18, 19]. These data reflected incomplete information of palatal shape since teeth position and inclination could have great impact on the measurements [18, 19]. In this study, palatal morphology was reconstructed up to the most inferior plane of maxillary alveolar bone [8], which can eliminate the influence of inclination of teeth. Furthermore, due to gender difference in 3D palatal morphology, the differences of 3D palatal morphology in vertical and sagittal patterns were compared in males and females separately in the present study.

### Comparison of 3D palatal morphology with different vertical patterns

In subjects with skeletal Class II and retrusive mandible, males with hyperdivergent and normodivergent showed narrower in the posterior part of palate. These results were in agreement with Parcha’s study, in which to explore the correlation between palatal morphology and skeletal pattern using GMM based on cephalograms and maxillary dental casts, and they reported skeletal Class II subjects with hyperdivergent pattern tended to have higher and narrower palate and emphasized that the most significant principal component related to palatal shape was vertical dimension [5].

According to the previous studies, it was speculated that one of the reasons to affect palatal morphology in different vertical patterns might be the function of masticatory muscles [20–22]. Lione et al. used ultrasound method to compare masticatory muscles in growing subjects among different vertical patterns and concluded that masticatory muscles of hyperdivergent subjects were relatively weak [21]. Besides, Kiliaridis et al. demonstrated that the weaker the function of masticatory muscles, the narrower maxillary dental arch might be [22]. Animal studies on growing rats also elucidated that decreased bone apposition on midpalatal suture and deficiency in transverse growth of maxilla was caused by decreased function of masticatory muscles [23]. All these results suggested that hyperdivergent subjects would be expected to have weak muscle function and narrow and high palatal shape [24]. Therefore, the clinician should pay attention to the exercise of muscles activity in hyperdivergent growing subjects during orthopedic maxillary expansion.

### Sagittal influence on 3D palatal morphology

In our study, the posterior part of palate in subjects with skeletal Class II and retrusive mandible was narrower than that of skeletal Class I subjects. Accumulated literatures reported the consistent results that palate of Class II subjects was narrower compared with Class I subjects [1, 25, 26]. Alarashi et al. analyzed maxillary base width with thin-plate spline analysis based on the cephalograms and revealed that constricted maxilla was associated with Class II subjects [1]. As for palatal height, our study observed the posterior part of palate in skeletal Class II subjects with retrusive mandible was flatter than that in skeletal Class I subjects of similar vertical pattern. However, Alarashi et al. reported the opposite result that Class II subjects had an increased palatal height when compared with Class I subjects [1]. The reasons leading to the different results might be contributed to the fact that vertical pattern was ignored in Alarashi’s study when palatal shape was compared among different sagittal patterns. According to Paoloni’s research, the most significant factor to affect palatal morphology was vertical pattern, and the higher the mandibular plane angle in skeletal Class II subject, the narrower and higher the palate was [4]. Furthermore, previous studies found that the proportion of hyperdivergent subjects in skeletal Class II with retrusive mandible was higher than normodivergent and hypodivergent subjects [27, 28]. Therefore, ignoring vertical factors might lead to inaccurate interpretation of the results. When discussing the differences of palatal morphology in our study between skeletal Class II subjects with retrusive mandible and skeletal Class I subjects, our conclusion was based on the similar vertical pattern, which might be more convincing, but still need further study.

## Conclusion

1. This study used a novel method to reconstruct 3D palatal morphology.
2. Palate morphology in males was higher and wider than females in the posterior part, regardless of different vertical and sagittal dimensions.
3. In skeletal Class II with retrusive mandible groups, males in hyperdivergent and normodivergent groups showed higher and narrower in the posterior part of the palate than that in hypodivergent group. Females in hyperdivergent and normodivergent groups also showed higher in posterior part of the palate but no obviously narrow compared with hypodivergent group.
4. In skeletal Class I groups, the posterior part of palate in hyperdivergent and normodivergent subjects showed higher but no significantly narrow compared with hypodivergent subjects for both genders.
5. Skeletal Class II subjects with retrusive mandible showed flatter and narrower in the posterior part of palate than that of skeletal Class I subjects.

## Data Availability

The datasets used and/or analysed during the current study are available from the corresponding author on reasonable request.

## Abbreviations

2D: Two-dimensional
3D: Three-dimensional
ANB: Angle formed by A-point–nasion–B-point
CBCT: Cone-beam computed tomography
DICOM: Digital imaging and communications in medicine
FH-MP: Angle between frankfort horizontal plane and mandibular plane
GMM: Geometric morphometric method
ICC: Intraclass correlation coefficient
NP-FH: Angle between the nasion-pogonion line and frankfort horizontal plane
PA: Palatal area
PH: Palatal height
PL: Palatal length
PV: Palatal volume
PW: Palatal width
RMSE: Root mean square estimate values
SD: Standard deviation
S-Go/N-Me: Ratio of the distance of sella-gonion to the distance of the nasion-menton
SNA: Angle formed by sella–nasion–A-point
SNB: Angle formed by sella–nasion–B-point
SN-MP: Angle between the sella-nasion line and mandibular plane
SPSS: Statistical package for social science
STL: Standard tessellation language
